# Endocrine Disrupting Effects of Triclosan on the Placenta in Pregnant Rats

**DOI:** 10.1371/journal.pone.0154758

**Published:** 2016-05-05

**Authors:** Yixing Feng, Pin Zhang, Zhaobin Zhang, Jiachen Shi, Zhihao Jiao, Bing Shao

**Affiliations:** 1 Beijing Key Laboratory of Diagnostic and Traceability Technologies for Food Poisoning, Beijing Center for Disease Control and Prevention, Beijing, China; 2 College of Urban and Environmental Sciences, MOE Laboratory for Earth Surface Processes, Peking University, Beijing, China; 3 Beijing Advanced Innovation Center for Food Nutrition and Human Health, College of Veterinary Medicine, China Agricultural University, Beijing, China; Xavier Bichat Medical School, INSERM-CNRS - Université Paris Diderot, FRANCE

## Abstract

Triclosan (TCS) is a broad-spectrum antimicrobial agent that is frequently used in pharmaceuticals and personal care products. Reports have shown that TCS is a potential endocrine disruptor; however, the potential effects of TCS on placental endocrine function are unclear. The aim of this study was to investigate the endocrine disrupting effects of TCS on the placenta in pregnant rats. Pregnant rats from gestational day (GD) 6 to GD 20 were treated with 0, 30, 100, 300 and 600 mg/kg/d TCS followed by analysis of various biochemical parameters. Of the seven tissues examined, the greatest bioaccumulation of TCS was observed in the placenta. Reduction of gravid uterine weight and the occurrence of abortion were observed in the 600 mg/kg/d TCS-exposed group. Moreover, hormone detection demonstrated that the serum levels of progesterone (P), estradiol (E2), testosterone (T), human chorionic gonadotropin (hCG) and prolactin (PRL) were decreased in groups exposed to higher doses of TCS. Real-time quantitative reverse transcriptase-polymerase chain reaction (Q-RT-PCR) analysis revealed a significant increase in mRNA levels for placental steroid metabolism enzymes, including UDP-glucuronosyltransferase 1A1 (UGT1A1), estrogen sulfotransferase 1E1 (SULT1E1), steroid 5α-reductase 1 (SRD5A1) and steroid 5α-reductase 2 (SRD5A2). Furthermore, the transcriptional expression levels of progesterone receptor (PR), estrogen receptor (ERα) and androgen receptor (AR) were up-regulated. Taken together, these data demonstrated that the placenta was a target tissue of TCS and that TCS induced inhibition of circulating steroid hormone production might be related to the altered expression of hormone metabolism enzyme genes in the placenta. This hormone disruption might subsequently affect fetal development and growth.

## Introduction

Triclosan (TCS) is a broad-spectrum antimicrobial agent that is used in clinical settings and in various personal care and consumer products, including soaps, hair products, toothpaste, medical devices, plastics, textiles, children toys, and others [1]. Currently, TCS is one of the more frequently detected and highly concentrated contaminants in aquatic and terrestrial environments [1]. TCS has been found in drinking water in certain regions of the USA and Canada [2]. According to a survey of U.S. streams and rivers, TCS was found in up to 57.6% of streams and rivers [3]. The detection frequency of TCS was 100% in urban catchments (with a maximum concentration of 61 ng/L) that cover approximately one-sixth of the land area of Singapore [4]. The concentrations of TCS in the receiving rivers of raw wastewater ranged from 11–98 ng/L in Switzerland [5]. In surface waters and sediment of the Pearl River system, China, the concentration of TCS ranged from 11–478 ng/L and 50–1330 ng/kg, respectively [6]. The main release of TCS into the environment results from personal care products containing approximately 0.1% to 0.3% (w/w) TCS [1]. Studies also have shown the prevalent exposure of the general population to TCS. TCS has been detected in many human matrices, including blood, urine, human milk, and amniotic fluid [7,8]. The National Health and Nutrition Examination Survey of 2003–2004 indicated that more than 70% of U.S. residents had detectible TCS in their urine [9]. TCS was detected in 100% of urine and 51% of cord blood samples in pregnant women in 181 expectant mothers from New York [10].

Analysis of the chemical structure of TCS implies that it may have chemical properties related to many toxic compounds, such as polychlorinated biphenyls (PCBs), polybrominated diphenyl ethers (PBDEs), bisphenol A (BPA) and dioxins, due to the similar halogenated biphenyl ether [11]. Data from biological studies have demonstrated that TCS might have endocrine disrupting effects in humans and other animals [12,13]. *In vitro* studies, TCS exhibited estrogenic and androgenic activity in both ER- and AR-mediated bioassays [14]. *In vivo* studies, TCS is also reported to have estrogenic activity as it can increase uterine weight in female rats [15], resulting in an earlier onset of vaginal opening [16], and can augment the level of vitellogenin in male fish [17]. Treatment with 18 and 27 mg TCS/animal/day disrupted blastocyst implantation on gestational days (GD) 1–3 as observed at GD 6, which again confirmed the estrogenic properties of TCS [18]. Conversely, an *in vitro* study reported that TCS exhibited anti-estrogenic activity in breast cells (MCF-7), inhibiting estradiol (E2)-induced cell growth [19]. In male rats, TCS exposure led to decreases in serum testosterone (T), sperm production and male accessory gland weight [20]. In addition, TCS could block thyroid hormone metabolism in pregnant rats [21] and affect thyroid hormone-mediated metamorphosis and postembryonic development in anuran [22,23]. In human choriocarcinoma JEG-3 cells, TCS exposure resulted in increased E2 and progesterone (P) secretion and decreased human chorionic gonadotropin (hCG) secretion [24].

The placenta is an important endocrine organ that connects the developing fetus to the maternal uterus. The placenta can synthesize a number of hormones, such as hCG, P and estrogen, which play important roles in implantation, pregnancy maintenance and embryo development [25,26]. Studies have shown that toxic and foreign chemicals may interfere with placental hormone secretion and further result in abortion, stunted fetal growth and intrauterine fetal death [27]. The chemical properties of TCS suggest its possible bioaccumulation and further environmental persistence [1]. However, as an endocrine-disrupting chemical, the potential effects of TCS on the placenta *in vivo* have not been studied. The aims of this study were to investigate the endocrine disrupting effects of TCS on the placenta in pregnant rats and to evaluate the mechanism of these effects by exploring the possible involvement of genes associated with hormone biosynthesis and decomposition.

## Materials and Methods

### Ethics statement

This study was carried out in strict accordance with the Guidelines for the Care and Use of Laboratory Animals of the National Institutes of Health. The protocol was approved by the Animal Care and Use Committee of Hubei Province (No. 00009367, 00021468).

### Animals

Sprague–Dawley (SD) rats aged 8–9 weeks (120 females and 60 males) were obtained from the Hubei Center for Disease Control and Prevention, Wuhan, China. Animals were maintained in a mass air displacement room with a 12 h light–dark cycle at 20–26°C and with a relative humidity of 30–70%. The animals had access to food and water *ad libitum*. All rats were acclimatized for 1 week before experiments were begun. Pairing for mating was 2 females:1 male. The females were examined each morning, and mating was confirmed by the presence of a sperm-positive vaginal smear; this day considered GD 0. Pregnant rats were housed one per cage and were randomly assigned to the control group or treatment groups (ten pregnant rats per group) in a manner that provided for comparable body weight, means and distribution across groups.

### Chemicals and treatments

TCS (CAS No. 3380-34-5, 99.7% purity) was purchased from Sigma Aldrich (St. Louis, MO, USA). To achieve different doses, TCS was dissolved in corn oil vehicle, which was prepared fresh each day. TCS was administered orally via gavage to rats in the treatment group for GD 6 to GD 20 at doses of 30, 100, 300 and 600 mg/kg body weight/day in a volume of 5 mL/kg of body weight. Control rats were treated similarly with the vehicle alone. Each experimental day, the rats were weighed to administer the dose per kilogram of body weight. The chosen doses were based on a study in which the acute oral 50% lethal dose (LD50) in SD rats was between 3,700 and > 5,000 mg/kg [28]. At GD 21, all rats were weighed and decapitated, in line with the euthanasia principle. Blood was collected and centrifuged at 3,000 rpm at 4°C for 15 min. Serum was stored at -80°C until analysis. Uteri (including placentas and fetuses) were weighed, and the number of live or dead fetuses was recorded. Then, placentas were immediately isolated, frozen in liquid nitrogen and stored at -80°C for RNA isolation and extraction.

### TCS concentration in tissue samples

The concentration of TCS in placenta was analyzed in 0, 30, 100 and 300 mg/kg/d TCS-exposed groups (placental concentration in the 600 mg/kg/d TCS-exposed group was absent due to the occurrence of abortion). In addition, in order to compare the cumulative amount of TCS in different tissues (including placenta, liver, kidney, ovary, adrenal, spleen, and fat), the samples of 30 mg/kg/d group were used. Tissue samples were homogenized in liquid nitrogen and hydrolyzed in lysis solution, including 2 mL acetic acid-sodium acetate (pH = 5.2) and 50 μL β–glucuronidase/arylsulfatase for 24 h at 37°C. Then, the samples were cooled to room temperature. In total, 40.0 ng TCS-13C^12^ (Item No. CLM-6779-MT-1.2) purchased from Cambridge Isotope Laboratories (Tewksbury, MA, USA) was added as internal standard solution. Then, the mixtures were extracted by 8 mL methanol for 20 min at 4°C and centrifuged at 3,000 × g for 10 min. The supernatants (800 μL) were diluted to 1 mL with pure water, and the concentrations of TCS were analyzed using an Acquity ultra performance liquid chromatography (UPLC) system coupled to a Xevo triple quadrupole mass spectrometer (Waters, Milford, MA, USA) [29,30].

### Serum hormone levels

Concentrations of serum luteinizing hormone (LH), follicle-stimulating hormone (FSH), hCG, prolactin (PRL), P, E2 and T were detected according to the manufacturers’ protocols by radioimmunoassay (RIA) using commercial kits from Beijing North Institute (Beijing, China). The sensitivities of the assays were 1.0 mIU/mL, 1.0 mIU/mL, 10 mIU/mL, 40 μIU/mL, 0.2 ng/mL, 2 pg/mL and 0.02 ng/mL for LH, FSH, hCG, PRL, P, E2 and T, respectively. The standard curve ranges for the assays were 5–200 mIU/mL, 2.5–100 mIU/mL, 50–1600 mIU/mL, 125–2000 μIU/mL, 0.2–100 ng/mL, 5–4000 pg/mL and 0.1–20 ng/mL for LH, FSH, hCG, PRL, P, E2 and T, respectively. The intra-assay and inter-assay coefficients of variation were less than 10% and 15%, respectively, for all the kits. Samples were analyzed and standard curves were generated simultaneously in all of the plates. Standards, controls and samples were analyzed in duplicate, and the average was calculated for each sample.

### RNA extraction and Q-RT-PCR

Three placentas (select randomly) from each pregnant rat were pooled, producing six pools of placenta for each group. Total RNA was isolated from the pools using an RNeasy Mini Kit (Qiagen, Hilden, Germany) according to the manufacturer’s instructions. Placental RNA in the 600 mg/kg/d TCS-exposed group was absent due to the occurrence of abortion. The total RNA was digested by DNase I (TaKaRa Biotechnology, Dalian, China) to remove genomic DNA contamination. The purified total RNA was measured at 260 and 280 nm using a Bio-Rad SmartSpec 3000 spectrophotometer (Bio-Rad, CA, USA). The 260:280 nm ratios and a 1% agarose-formaldehyde gel stained with ethidium bromide were used to verify the quality of the RNA in each sample. The first-strand cDNA was synthesized from 1 μg total RNA mixed with random 6-mers using a PrimeScript II 1st Strand cDNA Synthesis Kit (TaKaRa, Dalian, China). The expression levels of target genes were detected by real-time quantitative PCR, with the housekeeping gene β-actin used as an internal control. SYBR Green PCR Master Mix reagent kits (Applied Biosystems Inc., USA) were used according to the manufacturer’s instructions for quantification of gene expression with a 7300 real-time PCR system (Applied Biosystems Inc., USA). PCR primers (Table 1) were designed using Primer 5.0 software. The cycling conditions were as follows: 95°C for 10 s followed by 40 cycles of 95°C for 15 s and 60°C for 1 minute. After PCR, a melting curve analysis was performed to demonstrate the PCR product specificity. Every sample was analyzed in triplicate. The relative expression level of a target gene was presented as the sample versus the control [31].

**Table 1:** Sequences of primers used for Q-RT-PCR amplification.

Target Gene	GenBankAccession No.	Primer Sequences	Product Length (bp)	Tm (°C)
β-actin	NM_031144	FW:GTCCACCTTCCAGCAGATGT RW: GAAAGGGTGTAAAACGCAGC	73	60.0
CYP11A1	NM_017286	FW:GCCTCCAGACTTATTTCG RW: GTCTCGCTTCTGCCTTA	130	60.0
3βHSD1	M38178.1	FW:TGCCAGCCTTCATCTAC RW: CCTTCTCGGCCATCCTT	144	60.0
CYP17A1	NM_012753	FW:GAAGCCATCTCATTACACC RW: AGTAGCAAGGCCGTGAA	115	60.0
17βHSD3	NM_054007.1	FW:GACCGCCGATGAGTTTG RW: GGTGCTGCTGTAGAAGAT	133	60.0
CYP19A1	NM_017085	FW:CGTGGAGACCTGACAAA RW: GGATACTCTGCGATGAGA	116	60.0
UGT1A1	NM_012683	FW:TATTGGTGGGATAAACTGC RW: TTCCATCGCTTTCTTCT	142	60.0
SULT1E1	NM_012883.1	FW:AGGGAATTGTAGGAGAC RW: AGGGAATTGTAGGAGAC	81	60.0
SRD5A1	NM_017070	FW:CTCCTGGTCACCTTTGTC RW: GGTCACCCAGTCTTCAGC	105	60.0
SRD5A2	NM_022711	FW:CCTGTGCTTAGGGAAACC RW: CCACAAAGGAAGGCAACT	116	60.0
SRD5A3	NM_001013990	FW:TCTTGGGAATGATGATGTT RW: TGCTGGCAGTGGATGAC	109	60.0
PR	NM_022847	FW:TGCTTGCACGCTTGGAC RW: CAGGGAGATCGGTATTGG	86	60.0
AR	NM_012502	FW:AGGTTACGCCAAAGGGTT RW: GACAGTGAGGACGGGAT	96	60.0
ERα	NM_012689.1	FW:GACTCGCTACTGTGCTGTG RW: CGATGGTGCATTGGTTT	146	60.0

### Statistical analysis

All data analysis was performed with SPSS 13.0. The results are presented as the means ± SD (n = 6). Statistical analyses of hormone production and gene transcription profiles between control and exposed cells were evaluated by one-way analysis of variance (ANOVA) followed by post hoc LSD tests. A *p* value < 0.05 was considered statistically significant.

## Results

### Effects of TCS on net body weight gain and gravid uterine weight

**Fig 1:**
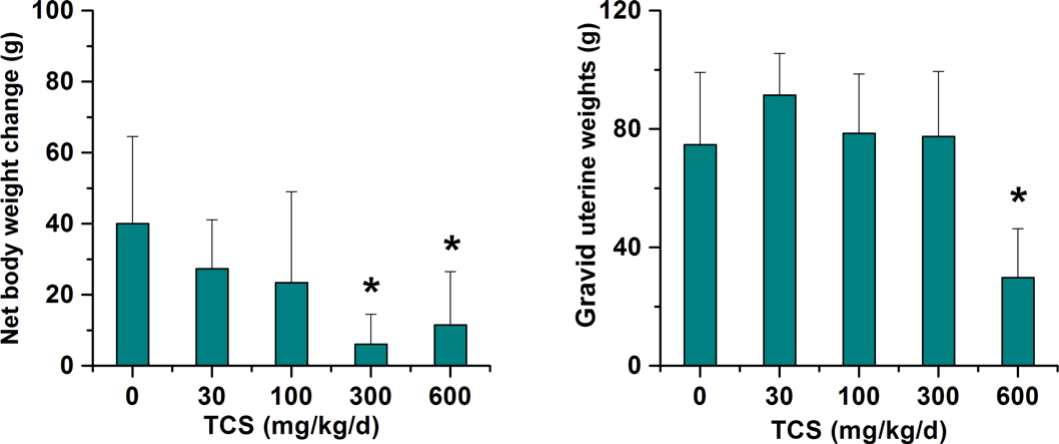
Net body weight gain and gravid uterine weight. Net body weight gain and gravid uterine weight of control and TCS-exposed pregnant rats (n = 6). For all panels, the bars represent the mean ± SD of six rats per group. Asterisks indicate a statistically significant difference compared to the vehicle control: *p < 0.05. Net body weight gain (GD 6–21) = body weight on GD 21 minus body weight on GD 6 and gravid uterine weight.

The net body weight gain and gravid uterine weight of pregnant rats exposed to TCS from GD 6 to GD 21 are shown in Fig 1. As gestation progressed, all groups gained body weight, but the weight gain was less in the 300 and 600 mg/kg TCS-exposed groups compared with the control group. The net body weight gain markedly decreased by 84.9% and 71.3% in the rats dosed with 300 and 600 mg/kg/d TCS, respectively (*p* < 0.05). Additionally, compared with the control group, significant differences in gravid uterine weight were observed in the 600 mg/kg/d TCS-exposed group (*p* < 0.05), whereas no significant differences were observed in the other three TCS treatment groups.

### The number of living fetuses

Litters were delivered by cesarean section on day 21 of pregnancy, and the number of living fetuses was calculated (Table 2). In the 0, 30, 100, 300 and 600 mg /kg/d TCS-exposed groups, the numbers of living fetuses were 93, 99, 87, 93 and 39, respectively.

**Table 2:** The number of living fetuses.

Group	Living fetuses
0 mg/kg/d	93
30 mg/kg/d	99
100 mg/kg/d	87
300 mg/kg/d	93
600 mg/kg/d	39

### TCS levels in different tissues

**Fig 2:**
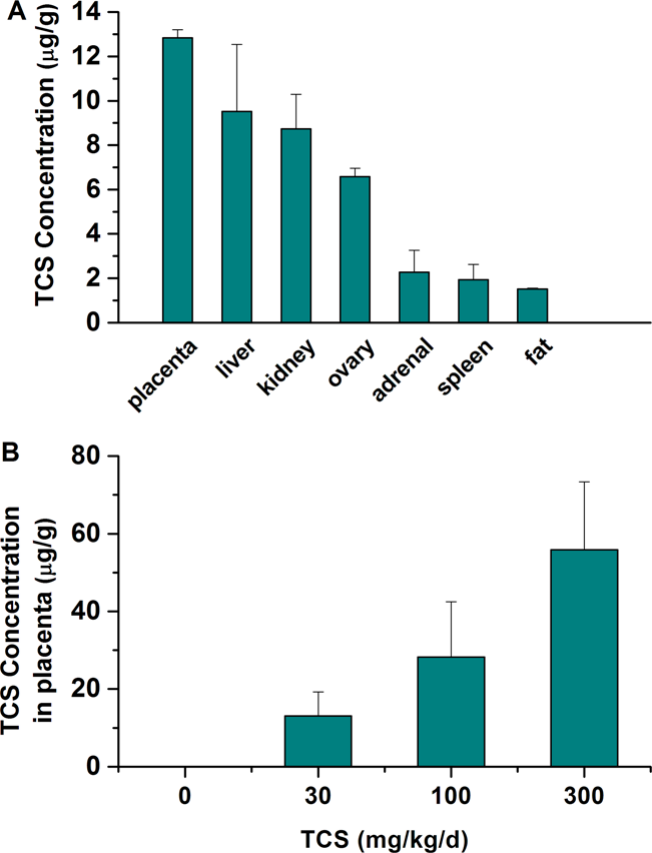
TCS concentrations in tissues. (A) Concentrations of TCS in different tissues from 30 mg/kg/d TCS-exposed pregnant rats (n = 6). (B) Concentrations of TCS in placenta from control and TCS-exposed pregnant rats. Bars represent the mean ± SD of six rats per group.

The concentrations of TCS in the different tissues of the 30 mg/kg/d TCS-exposed group are shown in Fig 2A. The average levels of TCS in the placenta, liver, kidney, ovary, adrenal, spleen and fat were 12.83, 9.52, 8.74, 6.58, 2.28, 1.93 and 1.51 μg/g, respectively. The concentrations of TCS in the placenta, liver and kidney were substantially higher than were those in the other tissues. The tissue distributions of TCS in order of concentration were the placenta > liver > kidney > ovary > adrenal > spleen > fat.

The concentrations of TCS in the placenta are shown in Fig 2B. The average levels of TCS in the placenta at the dose of 0, 30, 100 and 300 mg/kg/d TCS were 0.033, 13.05, 28.23 and 55.83 μg/g, respectively.

### Hormone levels in serum

**Fig 3:**
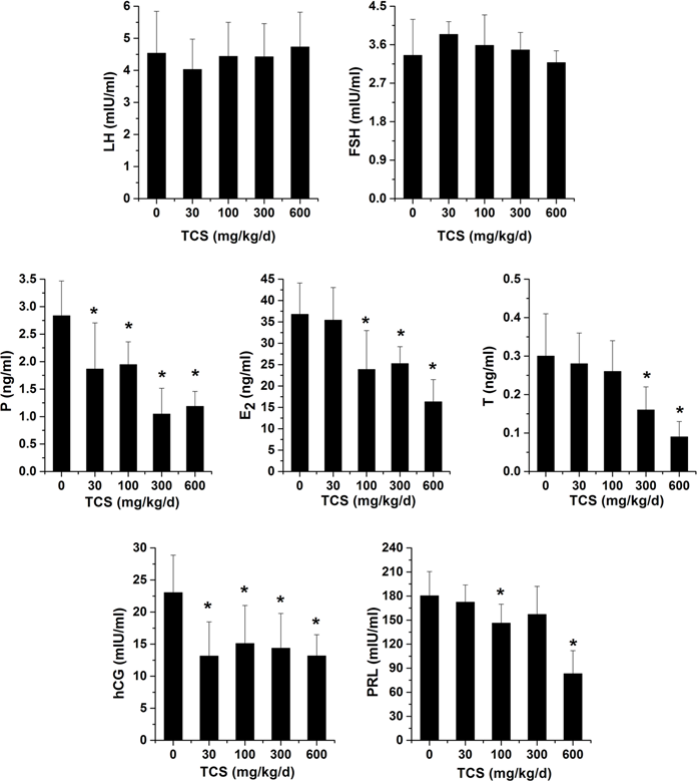
Hormone levels in serum. Serum levels of luteinizing hormone (LH), follicle stimulating hormone (FSH), progesterone (P), estradiol (E2), testosterone (T), human chorionic gonadotropin (hCG) and prolactin (PRL) from control and TCS-exposed pregnant rats. Bars represent the mean ± SD of six rats per group. Asterisks indicate a statistically significant difference compared with the vehicle control: *p < 0.05.

The effects of TCS on hormone secretion by the pituitary and placenta of pregnant rats are shown in Fig 3. The serum levels of LH and FSH secreted by the pituitary were identical across the entire exposure group compared with the control group (*p* > 0.05). However, the levels of hCG in serum exhibited significant inhibition in the TCS exposure groups compared with the control group: the levels of hCG were dramatically reduced by 43.0%, 34.4%, 37.4% and 42.6% in the 30, 100, 300 and 600 mg/kg/d TCS-exposed groups, respectively (*p* < 0.05). In rats receiving 100 and 600 mg/kg/d TCS, the levels of PRL in serum were dramatically decreased by 18.9% and 53.9%, respectively (*p* < 0.05), and no significant differences were observed between the control and other exposure groups (*p* > 0.05). In addition, the levels of P were significantly reduced by 34.3%, 31.4% and 63.3% and 58.3% in the 30, 100, 300 and 600 mg/kg/d TCS-exposed groups, respectively (*p* < 0.05). Similarly, in rats receiving 100, 300 and 600 mg/kg/d TCS, the levels of E2 in serum were dramatically decreased by 35.0%, 31.4% and 55.6%, respectively (*p* < 0.05). Additionally, the oral administration of 300 and 600 mg TCS resulted in 46.7% and 70.0% decreases in serum levels of T, respectively (*p* < 0.05).

### Expression levels of genes related to hormone biosynthesis and metabolism

**Fig 4:**
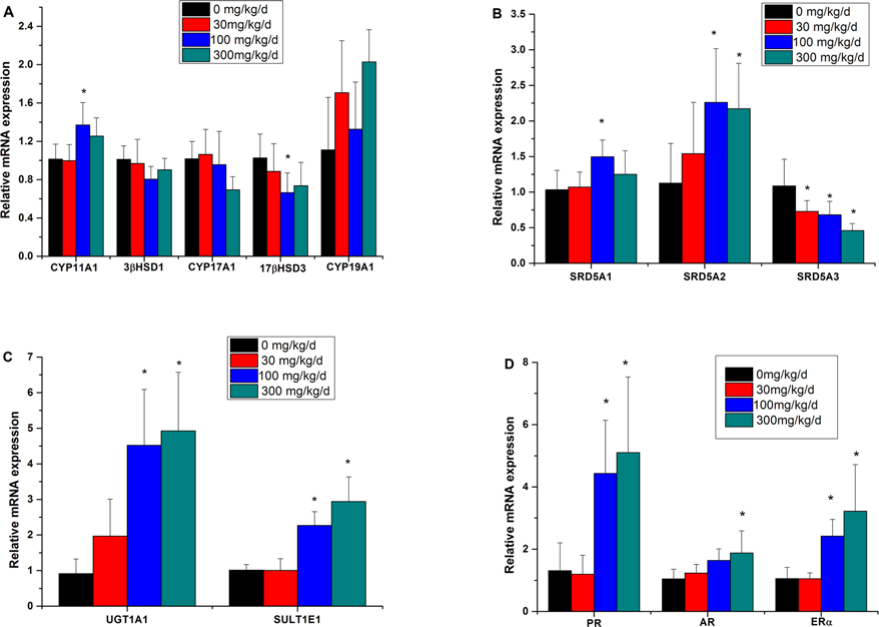
Relative expression levels of genes related to hormone biosynthesis and metabolism in the placenta. (A) Placental mRNA levels of CYP11A1, 3β-HSD1, CYP17A1, 17β-HSD3 and CYP19A1. (B) Placental mRNA levels of SRD5A1, SRD5A2 and SRD5A3. (C) Placental mRNA levels of UGT1A1 and SULT1E1. (D) Placental mRNA levels of PR, AR and ERα. The data represent the relative mRNA expression compared with the controls. Values are given as the mean ± SD of n = 18 (placenta) per group. *p < 0.05.

We studied the transcriptional levels of genes responsible for hormone (P, T and E2) biosynthesis and metabolism in the 0, 30, 100 and 300 mg/kg/d TCS-exposed placenta (Fig 4). As shown in Fig 4A, relative expression levels of genes responsible for hormone biosynthesis, including cytochrome P450 11A1 (CYP11A1), 3-beta hydroxysteroid dehydrogenase (3β-HSD1), cytochrome P450 17A1 (CYP17A1), cytochrome P450 19A1 (CYP19A1) and 17-beta hydroxysteroid dehydrogenase (17β-HSD3), were analyzed. CYP11A1 was significantly up-regulated in the 100 mg/kg/d TCS-exposed group (*p* < 0.05); no significant differences were observed in other TCS treatment groups compared with the control group. The expression levels of 3β-HSD1, CYP17A1, and CYP19A1 were not altered after TCS exposure, while expression of 17β-HSD3 was down-regulated in the 100 mg/kg/d TCS-exposed group (*p* < 0.05).

The genes involved in P and T metabolism are shown in Fig 4B. The mRNA expression levels of steroid 5α-reductase 1 (SRD5A1) and steroid 5α-reductase 2 (SRD5A2) were significantly increased in the 100 mg/kg/d and 300 mg/kg/d TCS-exposed groups compared with the control group (*p* < 0.05). However, the steroid 5α-reductase 3 (SRD5A3) gene was sharply down-regulated in all the TCS exposure groups (*p* < 0.05). Moreover, the genes related to E2 metabolism were investigated as shown in Fig 4C. UDP-glucuronosyltransferase 1A1 (UGT1A1) and estrogen sulfotransferase 1E1 (SULT1E1) were significantly up-regulated in rats exposed to 100 mg/kg/d and 300 mg/kg/d TCS (*p* < 0.05).

Hormones can trigger a number of events through binding to their receptors. In the present study, we further analyzed the expression levels of hormone receptor genes, including P receptor (PR), androgen receptor (AR) and estrogen receptor (ERα) (Fig 4D). The up-regulated mRNA expression levels of PR and ERα were observed in the 100 and 300 mg/kg/d TCS-exposed groups (*p* < 0.05). Similarly, the AR expression level was also increased in the highest dose group compared to that of the control group (*p* < 0.05).

## Discussion

Studies in mammals have shown that TCS is an endocrine-disrupting chemical [32] that has potential estrogenic or anti-androgenic effects [20,33]. The placenta is an important endocrine organ that can synthesize a number of hormones, which play important roles in pregnancy maintenance and embryo development [25,26]. An *in vitro* study demonstrated that TCS exposure could disturb hormone secretion by and cell viability of human choriocarcinoma-derived placental cells [24]. In addition, TCS easily accumulates in the placenta, which showed the greatest accumulation of TCS among the seven tested tissues. Therefore, considering the fact that studies of the placental toxicity of TCS *in vivo* are limited, the aims of this study were to investigate the endocrine disrupting effects of TCS on placenta and to further evaluate the potential mechanisms of these effects.

To the best of our knowledge, the present study is the first to examine the effects of TCS on the development and endocrine function of the placenta during rat pregnancy *in vivo*. The results showed that TCS treatment resulted in a reduction in body weight gain in the 300 and 600 mg/kg/d TCS-exposed groups and in a decrease in the number of living fetuses as well as miscarriage in the 600 mg/kg/d TCS-exposed group, which suggested that these doses of TCS might have general toxicity. Notably, the placenta displayed the greatest bioaccumulation of TCS among the seven examined tissues, and the cumulative amount of TCS in the placenta increased in a dose-dependent manner, suggesting that the placenta might be one of the target organs of TCS. Therefore, the potential hazards of TCS to human reproductive health, especially for pregnant women, require further examination.

Unlike the human placenta, which plays an important role in producing P and estrogen, the rat placenta does not produce estrogen, as rats do not express aromatase, and only secretes small amounts of P [34]. Indeed the rat placenta is the main source of T, and this T serves as the substrate for E2 synthesis in the corpus luteum [35]. Therefore, the rat placenta sustains the ovarian biosynthesis of E2, although it does not produce it [36]. The present study implied that a high dose of TCS has the potential to disturb hormone secretion in the placenta, including reductions in circulating P, E2 and T levels. To confirm whether the decreased hormone levels were related to the changing mRNA expression of enzymes related to steroid hormone synthesis, we analyzed the mRNA levels of CYP11A1, 3β-HSD1, CYP17A1, 17β-HSD3 and CYP19A1. Our results showed that the mRNA levels of these enzymes only slightly changed (increased CYP11A1 mRNA expression and reduced 17β-HSD3 mRNA expression were observed only in the 100 mg/kg/d TCS-exposed group) in the placentas of the TCS-exposed groups compared with those of the control, suggesting that the TCS-induced reduction in the levels of these hormones in pregnant rats might be independent of these enzymes. In addition to biosynthesis enzymes, metabolism enzymes are also involved in modulating hormone homeostasis. SULT1E1 and UGT1A1 catalyze the formation of E2 sulfonation and glucuronidation, respectively, which play key roles in estrogen metabolism during pregnancy in both mice and humans [37,38]. In this study, increased mRNA levels of UGT1A1 and SULT1E1 were observed in 100 and 300 mg/kg/d TCS-exposed placenta. Although the activity of these enzymes was not measured, the increased mRNA levels indicated their potential contributions to the reduced serum E2 levels in TCS-exposed pregnant rats. However, in sheep placenta, TCS was shown to be a very potent inhibitor of placental estrogen sulfotransferase activity [39]. Moreover, in the JEG-3 cells, TCS was found to increase E2 secretion, but the related mechanism was not investigated [24]. Thus, whether TCS is an inhibitor of E2 remains unclear and requires further investigation. In addition to entrogenic and anti-estrogenic effects, TCS shows anti-androgenic effects in male rats and rat Leydig cells. Following TCS exposure, decreased synthesis of T occurred due to reduced mRNA levels of steroidogenic enzymes in Leydig cells [40], and decreased serum levels of LH, FSH, P and T were observed in male rats [20]. In the present study, serum levels of T in the 300 and 600 mg/kg/d TCS-exposed groups and of P in all TCS treatment groups were dramatically decreased. However, the mRNA expression levels of steroid hormone synthesis enzymes in the TCS treatment group could not explain the decreased serum levels of T and P. Thus, we further detected the mRNA levels of SRD5A1 and SRD5A2, which are related to P and T metabolism. SRD5A1 and SRD5A2 can convert both P to 5α-dihydroprogesterone in human ovarian endometriosis [41] and T to 5α-dihydrotestosterone in human prostate [42], while the function of SRD5A3 in steroid metabolism is still unclear. Stiles and Russell found that SRD5A3 was not involved in steroid metabolism but played an important role in protein glycosylation [43]. Until now, the expression level of 5α-reductase and its function in the placenta have been unclear. In the present study, the mRNA expression level of SRD5A1 increased in the 100 mg/kg/d TCS-exposed group, and the mRNA expression levels of SRD5A2 were increased in the 100 and 300 mg/kg/d TCS-exposed groups. According to the results, we hypothesize that the increased mRNA expression levels of SRD5A1 and SRD5A2 might be attributed, at least in part, to the reduced serum levels of T and P.

Placental PRL hormone is important to fetal growth and pregnancy maintenance [44]. Gestational TCS exposure induced down-regulation of PRL hormone production in the 100 and 600 mg/kg/d TCS-exposed groups. In the placenta, trophoblast cells are the main source of PRL production and secretion, and the E2 and P levels are related to the proliferation and differentiation of these cells [45]. This study demonstrated that the serum levels of E2 and P were significantly decreased in the TCS-exposed groups. Consequently, disruption of these hormone levels may affect the development and differentiation of placental trophoblast cells in pregnant rats and further lead to changes in PRL secretion. During pregnancy, hCG is the first hormonal signal from the placenta to the mother and is mainly secreted by the placental syncytiotrophoblasts [46]. hCG plays an essential role in pregnancy maintenance by stimulating P and E2 steroidogenesis in the placenta [47,48]. Research has indicated that hCG can affect E2 production through alteration of aromatase expression and activity in the placenta [49]. In the present study, serum hCG levels in the 30 to 600 mg/kg/d TCS-exposed groups were significantly decreased and might have been associated with the decreased serum P and E2 levels. The related mechanism is not clear and requires further investigation.

Hormone receptors play important roles in the process of hormones exerting their biological effects. In pregnant women, increased PR and ER expression levels were observed in the endometrium and decidua after terminating early pregnancy by mifepristone application [50]. Similarly, higher total PR scores in canine endometrial stromal cells were found during resorption/abortion [51]. In human placenta, AR was found mainly in the syncytiotrophoblasts and stromal cells, and increased AR expression might be a possible mechanism for its association with preeclampsia [52]. Studies have noted an important role of androgen in placental vascular reactivity [53], which was related to fetal and placental size [54]. In addition, AR functions in the decidualization of human endometrial tissue [55]. In the present study, the mRNA expression levels of PR, AR and ERα were all up-regulated in response to higher dosage TCS treatments. Therefore, these data implied that TCS exposure might have adverse effects on the placenta and further increase reproductive health risks during pregnancy through altering the expression levels of hormone receptors. In contrast, the up-regulation of hormone receptors might function as a mechanism of protection from the possible deleterious effects of low hormone levels on the placenta and fetus.

In conclusion, the placenta might be a target tissue of TCS in pregnant rats. From GD 6-GD 20 in pregnant rats, TCS exposure decreased the circulating PRL, hCG, P, T and E2 levels in serum, which is a complex phenomenon. Based on the present results, we speculated that hormone metabolism enzymes, rather than biosynthesis enzymes, might contribute to the inhibition of P, T and E2 production after TCS exposure during pregnancy. Furthermore, we proposed that the increased expression of hormone receptors (PR, AR and ERα) in the placenta might be a protective mechanism to counteract the adverse effects that low hormone levels could have on the placenta and fetus.
